# A randomized controlled trial of intermittent Cervical Traction in sitting Vs. Supine position for the management of Cervical Radiculopathy

**DOI:** 10.12669/pjms.336.13851

**Published:** 2017

**Authors:** Rehan Ramzan Khan, Waqar Ahmad Awan, Sajid Rashid, Tahir Masood

**Affiliations:** 1 Dr. Rehan Ramzan Khan, DPT. Multan College of Physiotherapy, Multan Medical & Dental College, Multan, Pakistan; 2 Dr. Waqar Ahmad Awan, DPT. Isra institute of Rehabilitation Sciences, Isra University, Islamabad, Pakistan; 3 Dr. Sajid Rashid, DPT. Multan College of Physiotherapy, Multan Medical & Dental College, Multan, Pakistan; 4 Dr. Tahir Masood, PhD. Isra institute of Rehabilitation Sciences, Isra University, Islamabad, Pakistan

**Keywords:** Radiculopathy, Neck Pain, Posture

## Abstract

**Objective:**

To compare the effectiveness of intermittent cervical Traction in sitting vs. supine position for the management of cervical radiculopathy

**Methods:**

A randomized clinical trial was done to compare pain and disability modification of cervical radiculopathy patients by using cervical traction in sitting and supine positions. Forty patients (males and females aged between 18-60 years with chronic cervical radiculopathy) were recruited for the trial. Participants were randomized into two homogeneous groups by dice method. The Group-A (n=20) received 3-weeks of intermittent cervical traction in sitting position along with Transcutaneous Electric Nerve Stimulation (TENS) and hot pack. The Group-B (n=20) received the same treatment except the intermittent cervical traction that was applied in supine position. Participants were assessed two times: at baseline (week 0) and at the termination of rehabilitation (week 3). Neck disability index was used to collect the data before and after the treatment.

**Results:**

The mean age of the patients was 43.15±8.99 vs. 48.80±6.89 years in Group-A vs. Group-B respectively. Mean (±S.D.) weight of the patients was 74.75±12.11 vs. 74.60±11.24 kg in Group-A vs. Group-B respectively. Mean Neck Disability Index score at start of treatment was 30.30±7.46 vs. 30.75±7.85 in Group-A and Group-B respectively. There was a significant difference in Group-A and Group-B regarding aggregate NDI score at the end of treatment (19.45±7.12 vs. 11.05±4.40; p<0.0001).

**Conclusion:**

Supine position is better choice for applying cervical traction as compared to sitting position for the management of cervical radiculopathy comparing post interventional NDI score.

## INTRODUCTION

Cervical radiculopathy is an important subset of neck disorders, although less widespread than the common neck pain. Its severity in terms of pain and disability is much more as compared to general neck pain.^1^,^2^ It is a common condition, with annual incidence of around 83 per 100,000 and an increasing prevalence in the 5th decade of life (203 per 100,000).^3^ The patient’s specific cervical radiculopathy symptoms will depend primarily on which nerve is affected. The symptoms may also be referred to as radicular pain.^4^ Patients suffering from cervical radiculopathy often report neck pain. However, they seek medical attention mostly for disabling arm pain.^5^,^6^,^2^ Greater level of disability is experienced by those patients who suffer from arm pain combined with neck pain as compared to those patients who suffer only from neck pain.^2^

There are many interventional strategies for the management of cervical radiculopathy that ranges from conservative to operative. The conservative management includes immobilization by cervical collar, Pharmacotherapy by using Non steroidal anti inflammatory drugs, Physical therapy and manipulation and cervical traction. Home unit’s cervical traction may reduce symptoms of cervical radiculopathy.^7^ Cervical traction can be applied in either supine position 8,9 or by placing a patient in a halo vest in sitting position.^10^,^11^ The mode of cervical traction can be either continuous or intermittent.^12^ Maximum distraction of apophyseal joints depends on the combination of multiple factors including traction force, time and angle of cervical traction. Traction increases the intervertebral gap and so the space of intervertebral foramina to relieve pressure on the nerve root affected. The results of cervical traction are more satisfactory when severe muscle pain is subsided, and it should not be used in patients having symptoms of Myelopathy.^7^ Currently there is no agreement regarding best position for the application of cervical traction to manage cervical radiculopathy. The purpose of this study was to determine more effective position between sitting and supine for applying cervical traction to manage cervical radiculopathy.

**Fig.1:**
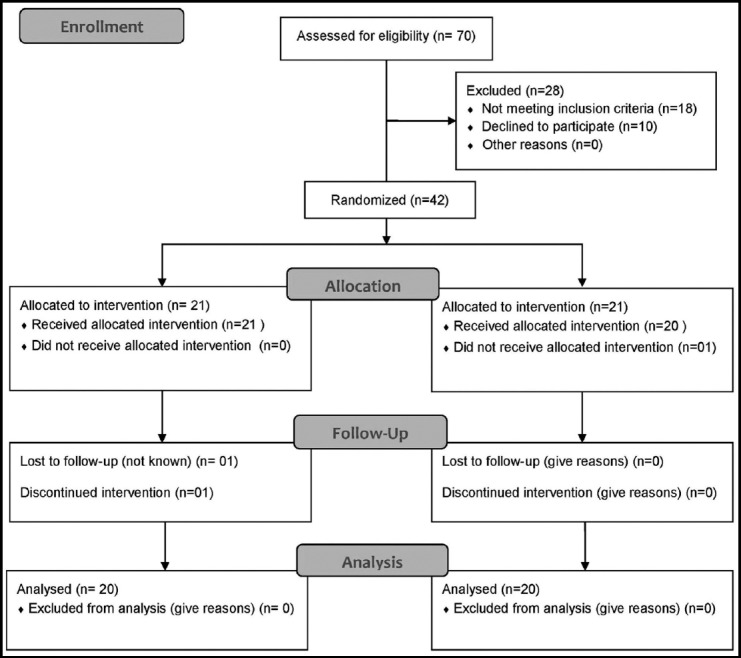
CONSORT diagram of the study.

## METHODS

A randomized clinical trial was done to compare pain and disability modification of cervical radiculopathy patients by using cervical traction in sitting and supine positions. Total 40 patients were recruited for this study. Both males and females aged between 18-60 years with chronic (after 4 weeks) cervical radiculopathy and the patients who were unresponsive to conservative medical treatment over a period of at least 04 weeks were included in this study. The patients with acute and sub acute cervical radiculopathy, advanced anatomical deformities of cervical spine, osteoporosis and unstable medical conditions such as cardiac disease and cancer were excluded. The diagnosis was made on the basis of patient’s history and physical examination. The physical tests used for diagnosis included Spurling test, shoulder abduction test, upper limb tension test and cervical range of motion. The participants were randomized into two homogeneous groups by throwing dice (e.g. below and equal to 3- Group A, over 3- Group B). The Group-A, (n=20) received 3-weeks of Electronic intermittent cervical traction (10% of Total Body Weight at 45 degree cervical flexion angle and 5 sessions per week with duration of 20 minutes per session for the application of cervical traction) in sitting position along with TENS and hot pack targeting the relief of radiculopathy symptoms, and the Group-B, (n=20) received the same three weeks of treatment except the intermittent cervical traction that was applied in supine position. Participants were assessed two times: at baseline (week 0) and at the termination of rehabilitation (week 3). Traction system ITO, TM-400 was used for applying cervical traction.

The study was conducted at Ibn e Siena Trust Hospital and Research institute. Permission was obtained from the Institutional Review Board and participants provided written consent before data collection. Convenience sampling technique was used to select the participants for this study. Standardized outcome measure Neck disability index was used to collect the data before and after the treatment. The questionnaire was translated into Urdu for better understanding. The researcher was present during form filling process to facilitate participants who were unable to read and write. Data was analyzed by using SPSS version 21. For presentation of categorical and demographic feature frequency percentage, mean and standard deviation were used. Paired samples t-test was used to analyze changes within the groups and independent samples t-test was used for differences between the groups. Alpha (α) level of significance was set at P value of 0.05.

## RESULTS

The mean age of the patients was 43.15±8.99 vs. 48.80±6.89 years in Group-A vs. Group-B respectively. Mean (±S.D.) weight of the patients was 74.75±12.11 vs. 74.60±11.24 kg in Group-A vs. Group-B respectively. Mean length of the disease was 15.5±7.7 vs. 22.95±10.86 months in sitting vs. supine position respectively (Table-I).

**Table-I:** Frequency distribution.

		*No. of Patients (%)*	

		*Group-A (SIT)*	*Group-B (SUP)*
Gender	Male	16(80%)	16(80%)
	Female	4(20%)	4(20%)
Weight (Kg)	41 –– 60	2(10%)	2(10%)
	61 –– 80	11(55%)	12(60%)
	81 –– 100	7(35%)	6(30%)
Chronicity (weeks)	6 –– 18	17(85%)	8(40%)
	19 –– 30	2(10%)	6(30%)
	31 –– 42	1(5%)	6(30%)
Sitting Hours (per day)	06 –– 08	7(35%)	6(30%)
	08 –– 10	10(50%)	8(40%)
	10 –– 12	3(15%)	6(30%)

Within group changes between 0 week and three week in Group-A and Group-B are shown in Table-II. The results in the both Groups A & B showed significant improvement after 3^rd^ week in individual items and as well as total NDI score.

**Table-II:** NDI Score in Both Groups (Within group comparison)

	*Group-A (SIT)*		*Group-B (SUP)*	

*Variable*	*(Week 0)*	*(Week 3)*	*(Week 0)*	*(Week 3)*

	*Mean± SD*	*Mean± SD*	*Mean± SD*	*Mean± SD*
Pain intensity	3.70±0.73	2.15±0.88	3.70±0.73	0.95±0.39
Personal care	3.45±0.89	2.25±0.91	3.35±0.93	1.05±0.60
Lifting	3.25±0.85	2.20±0.83	3.20±0.89	1.15±0.67
Reading	2.60±0.99	1.60±0.88	2.80±1.01	0.85±0.59
Headaches	2.60±1.05	1.70±0.92	2.60±0.88	0.85±0.49
Concentration	2.55±0.94	1.55±0.89	2.70±0.98	0.75±0.55
Work	3.20±0.62	2.20±0.62	3.30±0.66	1.40±0.68
Driving	3.20±0.77	3.25±0.85	3.25±0.85	1.45±0.51
Sleeping	2.65±0.99	1.65±1.04	2.75±0.97	1.15±0.67
Recreation	3.10±0.91	2.10±0.91	3.10±0.91	1.45±0.51
Total NDI	30.30±7.46	19.45±7.12	30.75±7.85	11.05±4.40

The groups differences at 0 week and three week in Group A & B are sown in Table-III. The results showed that both Groups (A & B) were comparable at 0 week. After three weeks, the result showed significant difference in results of both groups. Comparing the results of Group-A and Group-B, the supine position provided significantly better results in all domains of NDI except sleep (2.10±0.91 vs. 1.45±0.51, *p=0.08*) as compared to Group-A (Sitting).

**Table-III:** NDI Score in Both Groups (Between group comparison)

	*Week 0*			*Week 3*		

*Variable*	*Group A (SIT)*	*Group B (SUP)*	*p-value*	*Group A (SIT)*	*Group B (SUP)*	*p-value*
	
	*Mean±SD*	*Mean±SD*		*Mean±SD*	*Mean±SD*	
Pain intensity	3.70±0.73	3.70±0.73	1.00	2.15±0.88	0.95±0.39	0.00*
Personal care	3.45±0.89	3.35±0.93	0.73	2.25±0.91	1.05±0.60	0.00*
Lifting	3.25±0.85	3.20±0.89	0.85	2.20±0.83	1.15±0.67	0.00*
Reading	2.60±0.99	2.80±1.01	0.53	1.60±0.88	0.85±0.59	0.00*
Headaches	2.60±1.05	2.60±0.88	1.00	1.70±0.92	0.85±0.49	0.00*
Concentration	2.55±0.94	2.70±0.98	0.62	1.55±0.89	0.75±0.55	0.00*
Work	3.20±0.62	3.30±0.66	0.62	2.20±0.62	1.40±0.68	0.00*
Driving	3.20±0.77	3.25±0.85	0.84	3.25±0.85	1.45±0.51	0.00*
Sleeping	2.65±0.99	2.75±0.97	0.74	1.65±1.04	1.15±0.67	0.08
Recreation	3.10±0.91	3.10±0.91	1.00	2.10±0.91	1.45±0.51	0.00*
Total NDI	30.30±7.46	30.75±7.85	0.85	19.45±7.12	11.05±4.40	0.00*

* p<0.05

## DISCUSSION

There are several studies that have proved the effectiveness of cervical traction in patients with nerve root compression by alleviating pressure on nerve root as well as on the soft tissues.^13^,^14^ Cervical traction can be applied in ether supine position 8,15,9 or by placing a patient in a halo vest in sitting position.^10^, ^11^ The mode of cervical traction can be either continuous or intermittent.^12^ Maximum distraction of apophyseal joints depends on the combination of multiple factors including traction force, time and angle of cervical traction.

It is suggested that cervical traction provides more relief in conditions that involve nerve root compression by distracting intervertebral joints, removing pressure on intervertebral discs, enlarging intervertebral foramina, stretching soft tissues, limiting disc protrusion, freeing entrapped synovial membrane, releasing tethered nerve root and producing central vacuum to generate suctioning effect on protruded or herniated disc.^16^ Currently there is a lack of agreement among clinicians regarding the best postural position for the application of cervical traction to offer better improvement of symptoms of cervical radiculopathy.

Some clinicians think that supine position is better for the management of cervical radiculopathy because in this position the patient is relaxed more as compared to sitting position while others think sitting position is better one because it offer better distracting force needed to release pressure on compressed nerve roots.^10^,^11^ Colachs & strohm^15^ have reported that patient is more comfortable and soft tissues are more relaxed during supine position but Maitland^11^suggested that sitting position is effective one as it provides more glutei and lumbar support especially when slumped sitting position is assumed. The results of current study are in consensus with Colachs & strohm as supine position offered better relief of radiculopathy symptoms.

Dennis C W Fater et al. (2008) conducted a study to compare the distraction of cervical vertebra when traction is applied in supine lying and seated position. Cervical traction was applied to 17 asymptomatic volunteers in supine and seated position. Distances between the anterior and posterior borders of lower margin of C2 spine and upper margin of C7 spine were measured by a radiologist. There was a considerable increase in posterior vertebral space in supine position as compared to seated position.^17^ So the study concluded the supine position as a better one because there is more increase in intervertebral space to alleviate compression on trapped nerve roots as compared to sitting position cervical traction. In Current study supine position produced better clinical results as more force is translated for vertebral separation in supine position due to gravity elimination.

Another study was conducted by Deets D et al. to determine the position which provides greater posterior intervertebral separation during treatment with cervical traction. 14 kg and 18 kg weights were used in both sitting and supine position respectively with cervical angle 45 degree. Lateral radiograph of C4-C7 spine was taken and measurements done. There was greater increase in vertebral separation in supine position.^18^ The study concluded that supine position is more effective position for application of traction. The researcher also concluded that patient’s comfort and relaxation in supine position was the main cause for the increased intervertebral separation in supine position. Current study endorses the findings of this study and showed similar results regarding the effectiveness of supine position of cervical traction for the management of cervical radiculopathy manifestations.

Akinbo et al. (2013) did a study to check out cardiovascular response and negative effects of cervical traction application in sitting and in supine position; and also to compare the effectiveness of traction application in both sitting and supine position on pain and mobility of neck in patients with cervical Spondylosis.^19^ Out of twenty four patients, 9 patients of supine traction group and 15 patients of sitting traction group experienced adverse effects mostly cervical muscle tenderness. The study also disclosed the efficacy of two positions for the application of cervical traction in terms of pain and mobility of neck. The application of cervical traction in supine position showed a higher mean difference regarding pain reduction and neck mobility. The study concluded the effectiveness of positions, sitting and supine, for the alleviation of symptoms of cervical Spondylosis but supine position is a wiser and favorable choice. The results of current study are in agreement with the previous studies for the effectiveness of both sitting and supine position cervical traction for the management of cervical radiculopathy symptoms including cervical pain and stiffness.^9^,^12^,^20^ However, suggested supine position is a better one because soft tissues and muscles are less tensed in this position which favor greater vertebral separation to relive any compression on cervical spinal nerve roots provoking pain and radicular symptoms. The supine position is also more comfortable and easy to tolerate. Although positions, sitting and supine are both significantly effective for the improvement of cervical radiculopathy symptoms but the post-training comparison has proved the supine position to reduce the symptoms more than sitting position.

### Limitations of the study.

It did not include patients of cervical radiculopathy with traumatic origin and acute and sub acute onset. The participants recruited for study also represented a narrow range of ethnicity and time frame so generalizations should be made considering these limitations. A larger sample size with more diversity should be utilized and the study duration should be longer. The progress in the outcome should be assessed weekly for better evidence regarding the best position for cervical traction application to alleviate cervical radiculopathy symptoms.

## CONCLUSION

Both sitting and supine positions are effective for the improvement of cervical radiculopathy symptoms but the mean difference comparing the baseline and post interventional score is higher in supine position so supine position can be better choice for cervical traction as compared to sitting position for the management of cervical radiculopathy.

### Authors’ Contribution

**RRK** conceived, designed and did statistical analysis & editing of manuscript.

**RRK, WAA, SR & TM** did data collection and manuscript writing.

**TM** did review and final approval of manuscript.
